# Evolution and epidemic success of *Mycobacterium tuberculosis* in eastern China: evidence from a prospective study

**DOI:** 10.1186/s12864-023-09312-6

**Published:** 2023-05-05

**Authors:** Zonglei Zhou, Huaiming Yi, Qingrong Zhou, Luqi Wang, Yue Zhu, Weibing Wang, Zhengwe Liu, Haiyan Xiong

**Affiliations:** 1 School of Public Health, Fudan University, Shanghai, 200032 China; 2 Center for Disease Control and Prevention of Changshan County, 324200 Zhejiang, China; 3 Center for Disease Control and Prevention of Jiangshan City, 324100 Zhejiang, China; 4 Key Laboratory of Public Health Safety of Ministry of Education, Fudan University, Shanghai, 200032 China; 5 Institute of Tuberculosis Control, Zhejiang Provincial Center for Disease Control and Prevention, 310051 Zhejiang, China

**Keywords:** *Mycobacterium tuberculosis*, Drug resistance, Fitness compensation, Time-scaled haplotypic density, Epidemic success

## Abstract

**Background:**

Lineage distribution of *Mycobacterium tuberculosis* (*Mtb*) isolates is strongly associated with geographically distinct human populations, and its transmission can be further impacted by the bacterial genome. However, the epidemic success of *Mtb* isolates at an individual level was unknown in eastern China. Knowledge regarding the emergence and transmission of *Mtb* isolates as well as relevant factors may offer a new solution to curb the spread of the disease. Thus, this study aims to reveal the evolution and epidemic success of *Mtb* isolates in eastern China.

**Results:**

Of initial 1040 isolates, 997 were retained after removing duplicates and those with insufficient sequencing depth. Of the final samples, 733 (73.52%) were from Zhejiang Province, and 264 (26.48%) were from Shanghai City. Lineage 2 and lineage 4 accounted for 80.44% and 19.56%, with common ancestors dating around 7017 years ago and 6882 years ago, respectively. Sub-lineage L2.2 (80.34%) contributed the majority of total isolates, followed by L4.4 (8.93%) and L4.5 (8.43%). Additionally, 51 (5.12%) isolates were identified to be multidrug-resistant (MDR), of which 21 (29.17%) were pre-extensively drug-resistant (pre-XDR). One clade harboring *katG* S315T mutation may date back to 65 years ago and subsequently acquired mutations conferring resistance to another five antibiotic drugs. The prevalence of compensatory mutation was the highest in pre-XDR isolates (76.19%), followed by MDR isolates (47.06%) and other drug-resistant isolates (20.60%). Time-scaled haplotypic density analyses suggested comparable success indices between lineage 2 and lineage 4 (*P* = 0.306), and drug resistance did not significantly promote the transmission of *Mtb* isolates (*P* = 0.340). But for pre-XDR isolates, we found a higher success index in those with compensatory mutations (*P* = 0.025). Mutations under positive selection were found in genes associated with resistance to second-line injectables (*whiB6*) and drug tolerance (*prpR*) in both lineage 2 and lineage 4.

**Conclusions:**

Our study demonstrates the population expansion of lineage 2 and lineage 4 in eastern China, with comparable transmission capacity, while accumulation of resistance mutations does not necessarily facilitate the success of *Mtb* isolates. Compensatory mutations usually accompany drug resistance and significantly contribute to the epidemiological transmission of pre-XDR strains. Prospective molecular surveillance is required to further monitor the emergence and spread of pre-XDR/XDR strains in eastern China.

**Supplementary Information:**

The online version contains supplementary material available at 10.1186/s12864-023-09312-6.

## Introduction

In 2021, 10.6 million individuals were estimated to be infected with *Mycobacterium tuberculosis* (*Mtb*) worldwide, an increase of 4.5% since 2020 [1]. Among new cases, the estimated proportion of multidrug-resistant (MDR) or rifampicin-resistant (RR) tuberculosis (TB) was 3.6%, while the rate may reach up to 18% for those previously retreated [1]. Globally, China (14%) ranks second in the number of notified cases developing MDR/RR-TB only after India (27%) and followed by the Russian Federation (8%) [2]. Surveillance data in the past few years have revealed a substantial increase in the prevalence of MDR/RR-TB in China [3]. Growing evidence has suggested that drug-resistant *Mtb* strains account for the epidemic of TB [4, 5], and it is crucial to understand the molecular development of drug resistance. However, fewer data are available about the distribution of drug resistance and its evolutionary history in eastern China, since the new definitions for pre-extensively drug-resistant TB (pre-XDR-TB, MDR-TB with additional resistance to any fluoroquinolones) and XDR-TB (pre-XDR-TB with additional resistance to at least one of Group A drugs, including levofloxacin or moxifloxacin, bedaquiline, linezolid) were proposed by WHO at October 2020 [6].

Persistent spread of *Mtb* strains means the demand of considerable health care expenses, thus identifying strains with a higher risk of transmission is of great value to curb the epidemic. To our knowledge, several studies have explored the epidemic success and its correlates among *Mtb* strains using coalescent-based method and basic reproduction numbers from a perspective of population, like drug resistance and lineages [7, 8]. For the aggregated nature of these methods, they might mix the effects on transmission for isolates with distinct characteristics and fail to evaluate the transmission capacity for single strain [9]. Besides, performing further analyses based on smaller groups of isolates will greatly increase the estimation uncertainty of relevant factors for epidemic success [10]. By contrast, as an alternative approach, time-scaled haplotypic density (THD) analyses can calculate the success index for *Mtb* strains at an individual level and exhibit good performance in evaluating isolate-specific effects on epidemic success [9, 11]. By far, only fewer studies have investigated the epidemic success of *Mtb* strains and its risk factors at an individual level [5, 12], and relevant studies have not yet been performed in China. Furthermore, genomic factors contributing to the epidemic success of *Mtb* strains in eastern China have not been fully illustrated.

Thus, to offer further evidence about these gaps, whole genome sequencing (WGS) data of 997 *Mtb* isolates collected in eastern China were employed to ascertain the evolutionary history of population structure and acquisition of mutations conferring drug resistance. Additionally, we performed THD analyses to evaluate the epidemic success of *Mtb* strains and explored related genetic factors for the transmission.

## Methods

### Sample collection and genome sequencing

**Fig. 1:**
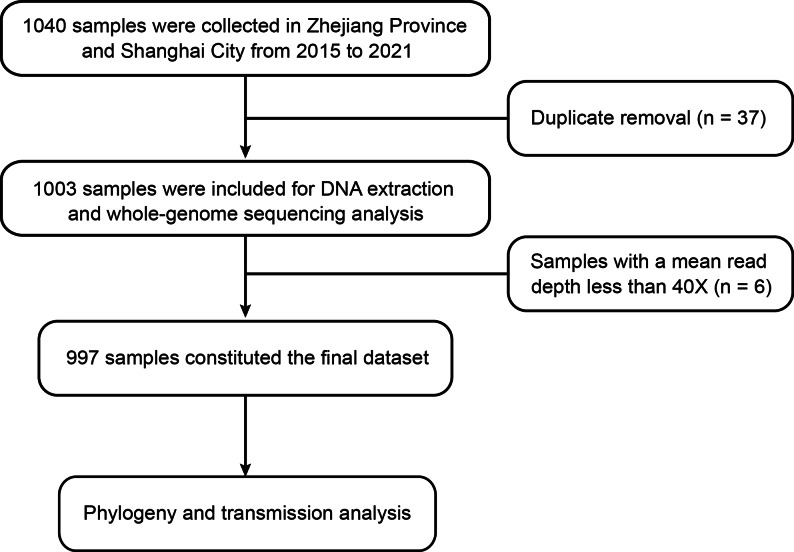
Study flow chart of sample collection and analyses

From 2015 to 2021, a total of 1040 samples were collected from culture-confirmed TB patients in eastern China, including 773 samples collected in two county hospitals in Zhejiang Province and 267 samples from two district hospitals in Shanghai City (Supplementary Fig. S1). After removing duplicates, 1003 samples remained to perform WGS, and 6 samples were eliminated due to insufficient sequencing depth (less than 40X). Of 997 *Mtb* strains included in the final analyses, 733 (73.52%) were collected in Zhejiang Province, and 264 (26.48%) were from Shanghai City (Fig. 1). Written informed consents were obtained from all participants.

The processes of WGS for *Mtb* strains were presented as follows: DNA extraction and fragmentation, repairing the end of DNA fragments, inducing sequencing adapters and index barcodes, purifying DNA fragments, PCR amplification, sequencing with Illumina HiSeq platform (https://www.illumina.com). After trimming unpaired reads and excluding low-quality sequence (using Trimmomatic version 0.36 [13]), reads were mapped to reference genome (GenBank accession number: NC_000962.3) using BWA-MEM version 0.7.17, followed by duplicate marking using SAMtools version 1.16.1 [14]. We employed BCFtools version 1.12 to call SNP and generated Variant Call Format (VCF) files [15]. SNP in PE/PPE/PGRS family gene, mobile genetic elements, phage sequence, and those linked with insertion and deletion regions were excluded using VCFtools version 0.1.16, and those with a minimum mapping quality of 30, a minimum base quality score of 20, a read depth from 10 to 2000, and a minimum variant frequency of 95% were retained [16].

### Lineage and drug resistance prediction

To accurately ascertain the phylogeny of *Mtb* isolates, we also removed SNP associated with drug resistance beside those excluded as described above. Thereby, a concentrated SNP alignment with 73,802 sites was obtained for further analyses. Specifically, a maximum-likelihood (ML) phylogenetic tree was constructed using IQtree software version 2.2.0 [17] with the modelFinder option. To test the confidence of phylogeny, we implemented bootstrap analysis using the ultrafast bootstrap approximation with 1000 replicates. The output tree file was visualized and annotated using the online tool iTOL version 6 (https://itol.embl.de). Besides, cross-checks were performed to ensure optimum lineage allocation for *Mtb* strains based on the results of TB-Profiler version 4.1.1 [18].

Variants in 38 genes involving the mechanism of resistance to the first- and second-line drugs were used to predict the resistance profile of *Mtb* strains, which were further checked by TB-Profiler version 4.1.1 [18]. Additionally, the compensatory mutations in *rpoA* and *rpoC* genes [19] for RR isolates, and variants in *ahpC* upstream region for isoniazid-resistant (HR) isolates [20] were assessed (Supplementary Table 1). In this study, MDR-TB was defined when *Mtb* strains were both HR and RR. According to the updated definition suggested by WHO [6], pre-XDR-TB referred to MDR/RR-TB which were additionally resistant to any fluoroquinolones, and XDR-TB refers to pre-XDR-TB with resistance to at least one of Group A drugs.

### Reconstruction of time-scaled phylogeny

To explore the evolution and chronological order of occurrence of genetic drug resistance and compensatory mutations for *Mtb* strains in eastern China, Bayesian Markov chain Monte Carlo (MCMC) algorithm was implemented using BEAST software version 1.10.4 [21] to estimate the time to the most recent common ancestor (tMRCA) and corresponding 95% highest posterior density (HPD). For the Bayesian MCMC model, we assumed an uncorrelated relaxed molecular clock with normal distribution and a mean tMRCA of 6897 years ago with a SE (standard error) of 200 years for L2.2 isolates in Zhejiang Province reported in a previous study [22]. The GTR + Γ4 model was selected for Bayesian-based coalescent analyses, suggested by jModeltest software version 2.1.7 [23].

To ensure reliable results, we ran five MCMC chains with a total of 1⋅10^8^ generations, sampling every 5000 generations. LogCombiner software version 1.10.4 was employed to combine log files and tree files generated from each MCMC chain. We employed Tracer software version 1.7.2 to assess model convergence status by checking the value of effective sample size (ESS), which was greater than 200 representing a good convergence. After discarding 10% of initial trees as burn-in, Bayesian skyline plot was generated by Tracer software version 1.7.2 and visualized using R software version 4.0.1.

### Epidemic success analyses

In the presented study, snp-dists version 0.8.2 (https://github.com/schultzm/snp-dists) was used to obtain the matrix of pairwise SNP distance, which was further used to calculate THD success index to evaluate the epidemic success status of *Mtb* strains as described elsewhere [11]. The analysis was carried out using *thd* package (https://github.com/rasigadelab/thd) in R software version 4.0.1. Specifically, the following user-defined parameters were used, including a mean evolutionary rate of 1⋅10^− 7^ mutations per genome per site, an effective genome size of 4⋅10^6^ (number of loci for SNP calling), and a time scale of 200 years to reflect the transmission of *Mtb* strains [11].

### Homoplasy analyses

To detect identical SNP occurring in phylogenetically unrelated *Mtb* isolates of lineage 2 and lineage 4, homoplasy analysis was performed using *HomoplasyFinder* package [24] and R software version 4.0.1. For data input, SNP in genes indicating drug resistance and fitness compensation were reintroduced to generate concatenated alignments, and ML phylogenetic trees were created using SNP alignments without genes related to drug resistance and bacterial fitness.

### Statistical analyses

Descriptive analyses were performed for sampling region, lineage distribution, drug resistance, and compensatory mutations of *Mtb* strains. Between-group comparison for category variables was conducted using chi-square test, or Fisher’s exact test if the expected value was smaller than 5. Kruskal-Wallis H test was performed to detect the difference in THD distribution across groups. In this study, we attempted to investigate the risk factors for the epidemic success of *Mtb* strains in an exploratory nature, thus no multivariate linear regression analysis adjusting for cofounders was carried out. The resistance pattern of *Mtb* isolates was further categorized as: sensitive, non-MDR, MDR, pre-XDR, where non-MDR included HR and RR *Mtb* strains, and those with other drug resistance except the above categories. R software version 4.0.1 was used to conduct statistical analyses and create plots. A two-tailed *P* value of less than 0.05 indicated statistical significance.

## Results

### Population phylogeny

**Fig. 2:**
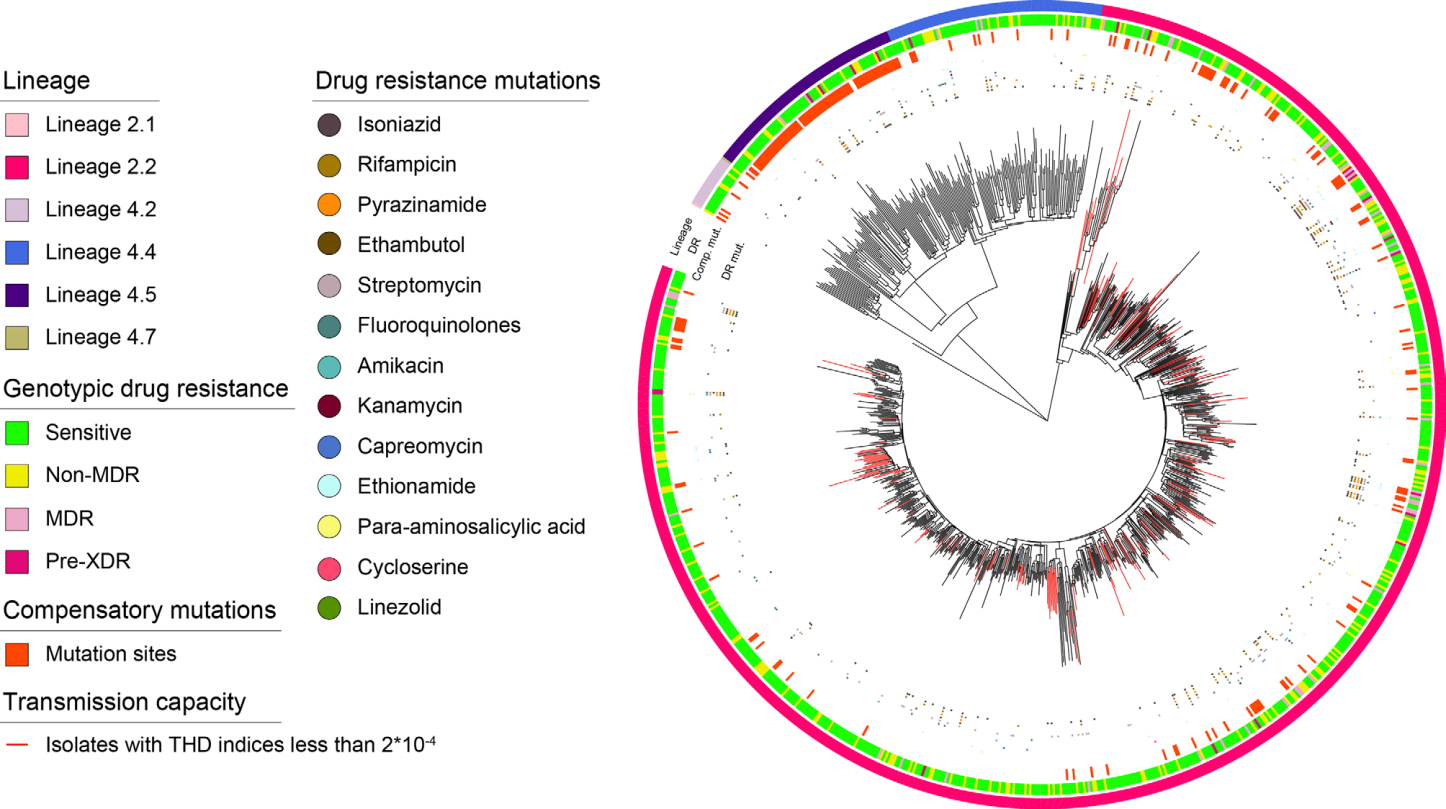
Maximum-likelihood phylogenetic tree of 997 *Mtb* strains in eastern China, 2015–2021. From outer to inner: lineages, drug resistance, compensatory mutations, drug resistance-associated mutations. Branches in red represent isolates with THD indices less than 2⋅10^− 4^

Of 997 strains included in our analyses, the average read depth was 279.70±122.07X, and the mean breadth of coverage was 98.81±1.21%. To illustrate the population structure of *Mtb* strains circulating in eastern China, a ML evolutionary tree was created (Fig. 2). As shown in the phylogenetic tree, 802 (80.44%) strains were identified as lineage 2, 195 (19.56%) strains belonged to lineage 4. For lineage 2, L2.2 was the predominant sub-lineage (99.88%), followed by L2.1 (0.12%). Besides, L2.2 also accounted for the majority of total strains (80.34%). Lineage 4 was found to comprise four sub-lineages, the frequency of which from high to low were L4.4 (45.64%), L4.5 (43.08%), L4.2 (10.77%) and L4.7 (0.51%). The results of between-group comparison analysis did not indicate a significantly different distribution of lineages between Zhejiang Province and Shanghai City (*χ*^2^ = 0.56, *P* = 0.454) (Table 1).

**Fig. 3:**
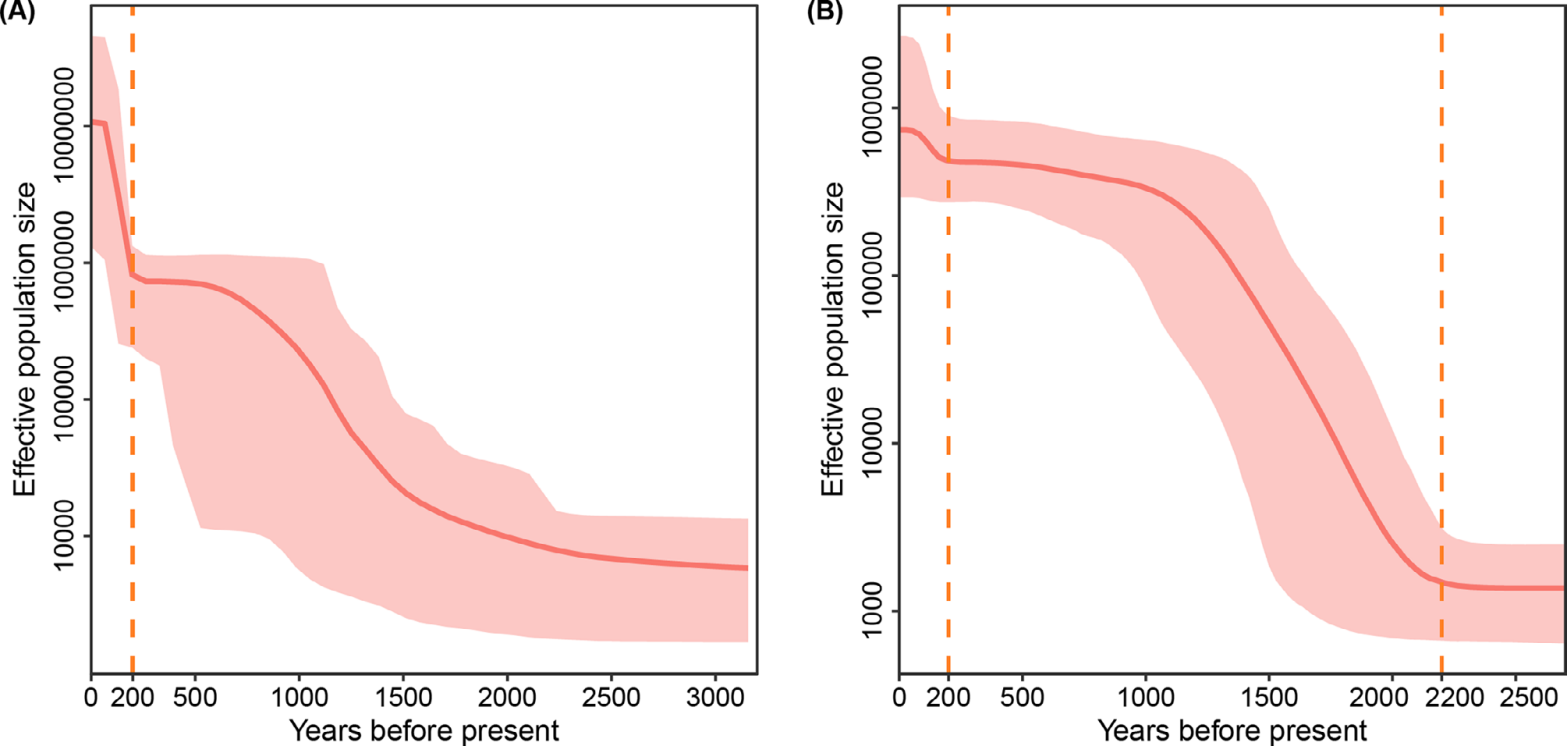
Bayesian skyline plot indicating the changes in population size over time for lineage 2 (A) and lineage 4 (B) in eastern China. Solid lines represented the mean value of effective population size with shaded areas representing the corresponding 95% highest posterior density

Bayesian phylogenetic analysis suggested the mean tMRCA of lineage 2 was 7017 years ago (95% HPD, 6555–7469), and the tMRCA for lineage 4 was estimated to be 6882 years ago (95% HPD, 5423–8487). For the sub-lineages, L4.2 was detected to emerge around 4565 years ago (95% HPD, 1065–8276), followed by L4.5, which first emerged around 3768 years ago (95% HPD, 2411–6817), and L4.4 was found as the most modern sub-lineage appearing around 2430 years ago (95% HPD, 1340–3311) (Table 2). The results of coalescent-based demographic reconstructions suggested an increased population size of lineage 2, especially since 200 years ago, and lineage 4 was predicted to experience a notable expansion around 200 and 2200 years ago, respectively (Fig. 3).

**Table 1:** Drug resistance of *Mtb* strains stratified by lineages and compensatory mutations in eastern China

Drug resistance	Overall	Lineages		*P*	Compensatory mutations		*P*
		Lineage 2	Lineage 4		0	≥1	
Overall (n, %)	997 (100.00)	802 (80.44)	195 (19.56)	-	793 (79.54)	204 (20.46)	-
Sampling region				0.454			0.131
Zhejiang	733 (73.52)	585 (79.81)	148 (20.19)		592 (80.76)	141 (19.24)	
Shanghai	264 (26.48)	217 (82.20)	47 (17.80)		201 (76.14)	63 (23.86)	
Susceptibility profile (n, %)				0.375^*^			< 0.001^*^
Sensitive	726 (72.82)	577 (79.48)	149 (20.52)		603 (83.06)	123 (16.94)	
Non-MDR	199 (19.96)	164 (82.41)	35 (17.59)		158 (79.40)	41 (20.60)	
MDR	51 (5.12)	45 (88.24)	6 (11.76)		27 (52.94)	24 (47.06)	
Pre-XDR	21 (2.11)	16 (76.19)	5 (23.81)		5 (23.81)	16 (76.19)	
Drug resistance (n, %)							
INH-resistant	152 (15.25)	121 (79.61)	31 (20.39)	0.864	93 (61.18)	59 (38.82)	< 0.001
RFP-resistant	89 (8.93)	77 (86.52)	12 (13.48)	0.169	45 (50.56)	44 (49.44)	< 0.001
EMB-resistant	66 (6.62)	54 (81.82)	12 (18.18)	0.896	31 (46.97)	35 (53.03)	< 0.001
PZA-resistant	46 (4.61)	39 (84.78)	7 (15.22)	0.569	18 (39.13)	28 (60.87)	< 0.001
SM-resistant	123 (12.34)	107 (86.99)	16 (13.01)	0.067	76 (61.79)	47 (38.21)	< 0.001
FLQ-resistant	61 (6.12)	47 (77.05)	14 (22.95)	0.601	39 (63.93)	22 (36.07)	0.003
AM-resistant	14 (1.40)	13 (92.86)	1 (7.14)	0.326^*^	7 (50.00)	7 (50.00)	0.013^*^
KM-resistant	8 (0.80)	6 (75.00)	2 (25.00)	0.658^*^	4 (50.00)	4 (50.00)	0.060^*^
CM-resistant	8 (0.80)	7 (87.50)	1 (12.50)	1.000^*^	6 (75.00)	2 (25.00)	0.670^*^
ETO-resistant	36 (3.61)	28 (77.78)	8 (22.22)	0.844	19 (52.78)	17 (47.22)	< 0.001
PAS-resistant	10 (1.00)	8 (80.00)	2 (20.00)	1.000^*^	8 (80.00)	2 (20.00)	1.000^*^

Note: *Fisher’s exact test. INH, isoniazid; RFP, rifampicin; EMB, ethambutol; PZA, pyrazinamide; SM, streptomycin; FLQ, fluoroquinolones; AM, amikacin; KM, kanamycin; CM, capreomycin; ETO, ethionamide; PAS, para-aminosalicylic acid

**Table 2:** The time to the most recent common ancestor for lineage 2, lineage 4 and sub-lineages in eastern China

Statistics	Lineage 2	Lineage 4	L4.2	L4.4	L4.5
Mean (tMRCA)	7017	6882	4565	2430	3768
SE of the mean	69.34	311.54	168.21	353.72	551.60
Median (tMRCA)	7009	6845	5690	2218	3348
Geometric mean	7013	6823	3534	2325	3592
95% HPD	6555–7469	5423–8487	1065–8276	1340–3311	2411–6817

### Evolution of drug resistance

Of 997 *Mtb* isolates, 21 (2.11%) were defined to be pre-XDR, 51 (5.12%) were defined to be MDR according to the new definition of drug resistance proposed by WHO [6], and drug-sensitive *Mtb* strains accounted for 72.82% of all samples. However, we did not detect mutations previously reported to be in association with resistance to the newly introduced drugs, bedaquiline and delamanid, as well as the WHO Group A drug, linezolid [25–28]. Notably, we found a high prevalence of resistance mutations against first-line drugs among *Mtb* isolates circulating in eastern China, especially for isoniazid (15.25%) and streptomycin (12.34%). As for second-line drugs, the resistance rate was the highest for fluoroquinolones (6.12%), followed by ethionamide (3.61%), amikacin (1.40%), para-aminosalicylic acid (1.00%), kanamycin (0.80%) and capreomycin (0.80%). No significant difference in drug resistance rate was shown between lineage 2 and lineage 4 (Table 1). Among all main sub-lineages (L2.2, L4.4, and L4.5), the highest resistance rate was detected for isoniazid. Besides, there was a high level of resistance to streptomycin in L2.2 and L4.5, but relatively lower than fluoroquinolones in L4.4 (Supplementary Fig. S2).

**Fig. 4:**
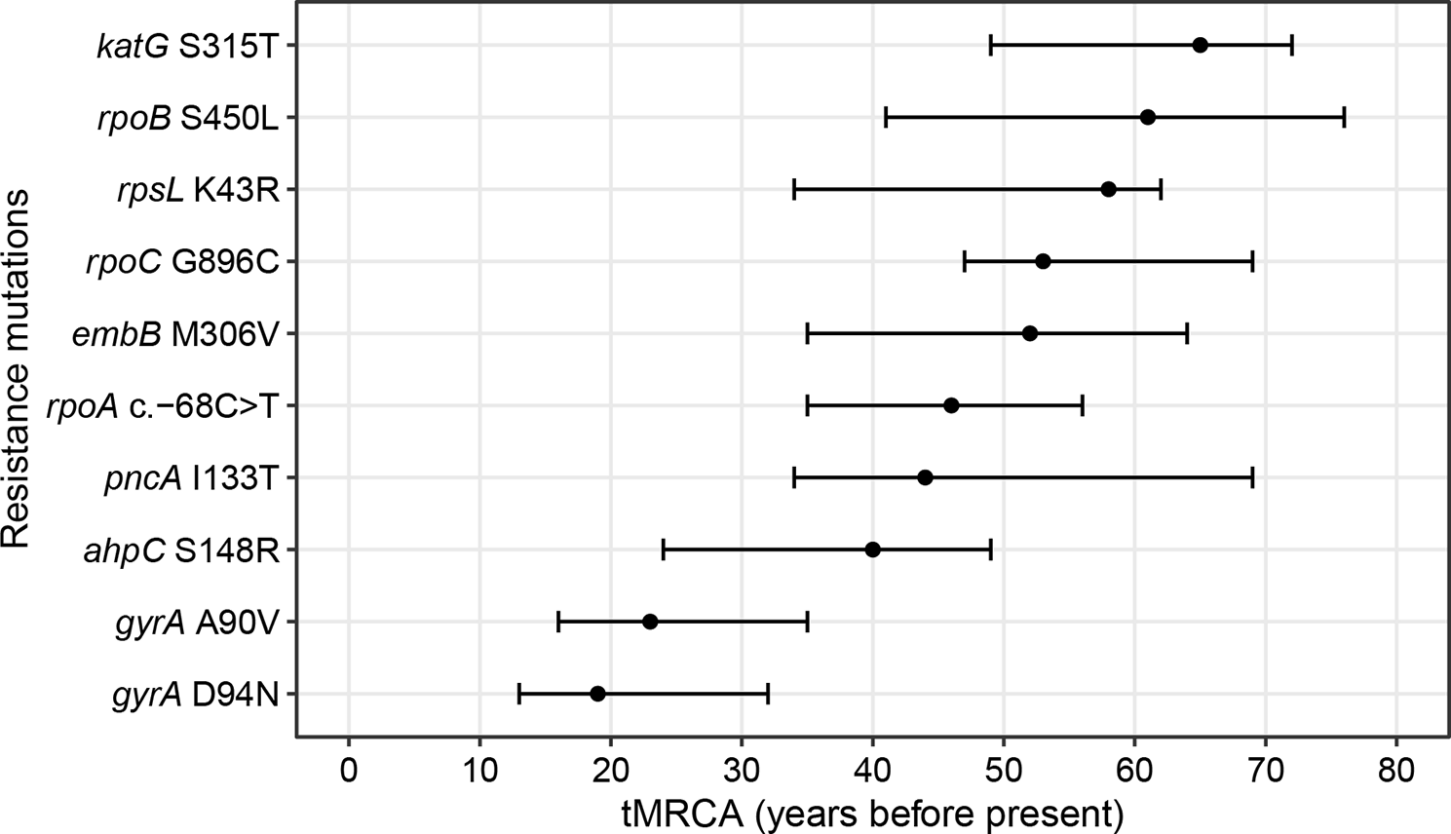
The time to the most recent common ancestor of clades acquiring drug-resistant mutations and compensatory mutations. *rpoC* G896C, *rpoA* c.-68 C > T, *ahpC* S148R predict compensatory effects. Dot represented the mean estimated tMRCA with error bar representing the corresponding 95% highest posterior density

Bayesian evolutionary analyses revealed that isoniazid resistance (*katG* S315T) first emerged in eastern China around 65 years ago (95% HPD, 49–72), followed by rifampicin resistance (*rpoB* S450L) around 61 years ago (95% HPD, 41–76). Isolates acquired resistance to pyrazinamide (*pncA* I133T), ethambutol (*embB* M306V), and streptomycin (*rpsL* K43R) around 44 (95% HPD, 34–69), 52 (95% HPD, 35–64) and 58 years ago (95% HPD, 34–62), respectively. Mutations conferring resistance to fluoroquinolones, including *gyrA* A90V and *gyrA* D94N, were estimated to appear around 23 (95% HPD, 16–35) and 19 (95% HPD, 13–32) years ago. The time of acquisition of putative compensatory mutations, *rpoA* c.-68 C > T, *rpoC* G896C, and *ahpC* S148R was estimated around 46 (95% HPD, 35–56), 53 (95% HPD, 47–69) and 40 (95% HPD, 24–49) years ago, respectively (Fig. 4).

### Impacts of genomic factors and drug resistance on epidemic success

**Fig. 5:**
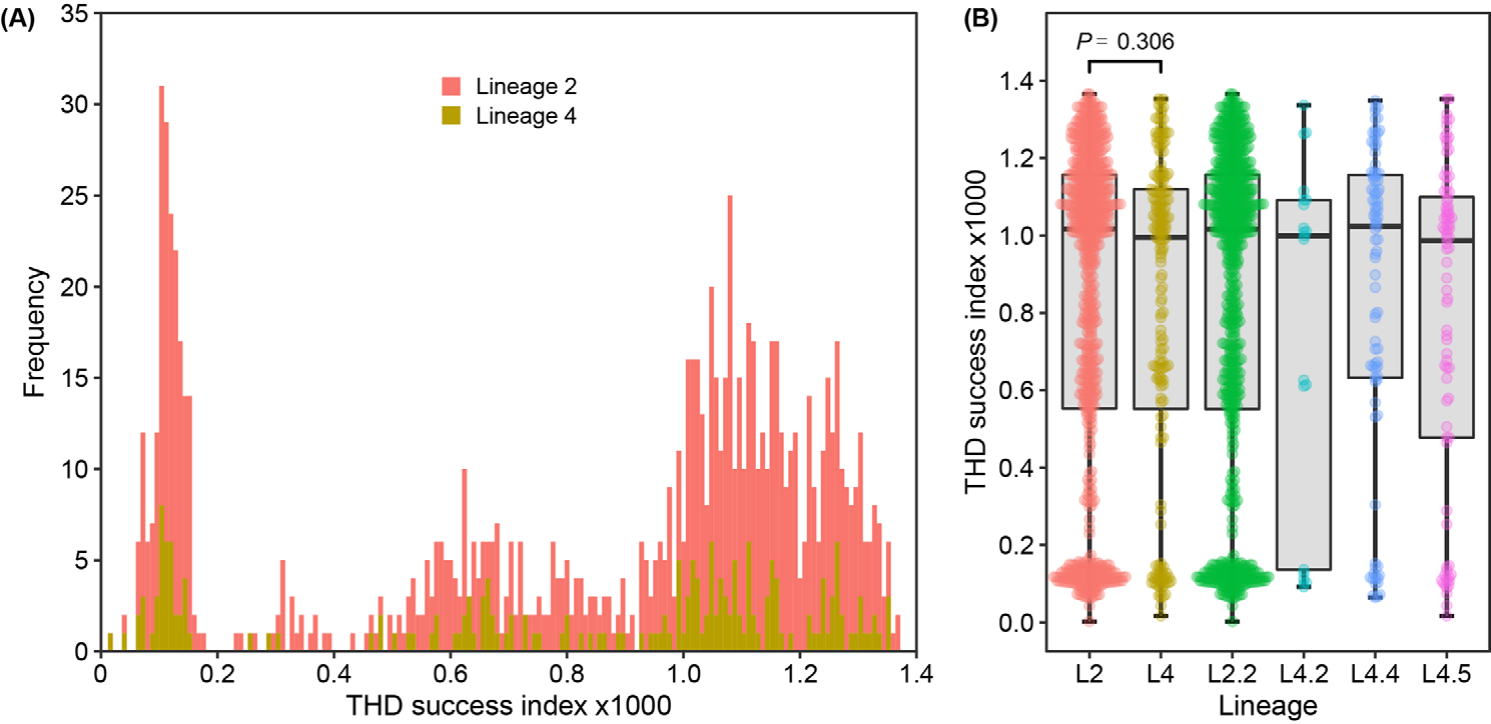
THD success index for lineage 2, lineage 4 and main sub-lineages

Firstly, we compared the THD success index across lineages and main sub-lineages using Kruskal-Wallis H test. The results demonstrated comparable THD indices between lineage 2 and lineage 4 (*P* = 0.306), the medians of which were 1.02 (IQR, 0.55–1.16) and 1.00 (IQR, 0.55–1.12), respectively. The median THD indices for sub-lineage L2.2, L4.2, L4.4 and L4.5 were calculated to be 1.02 (IQR, 0.55–1.16), 1.00 (IQR, 0.14–1.09), 1.02 (IQR, 0.63–1.16) and 0.99 (IQR, 0.48–1.10), respectively (Fig. 5). Furthermore, given the bimodal distribution of THD indices of L2.2, we employed a cutoff value of 2⋅10^− 4^ to identify isolates with low transmission capacity and found irregular positions of isolates with low THD indices in the phylogenetic tree (Fig. 2).

**Fig. 6:**
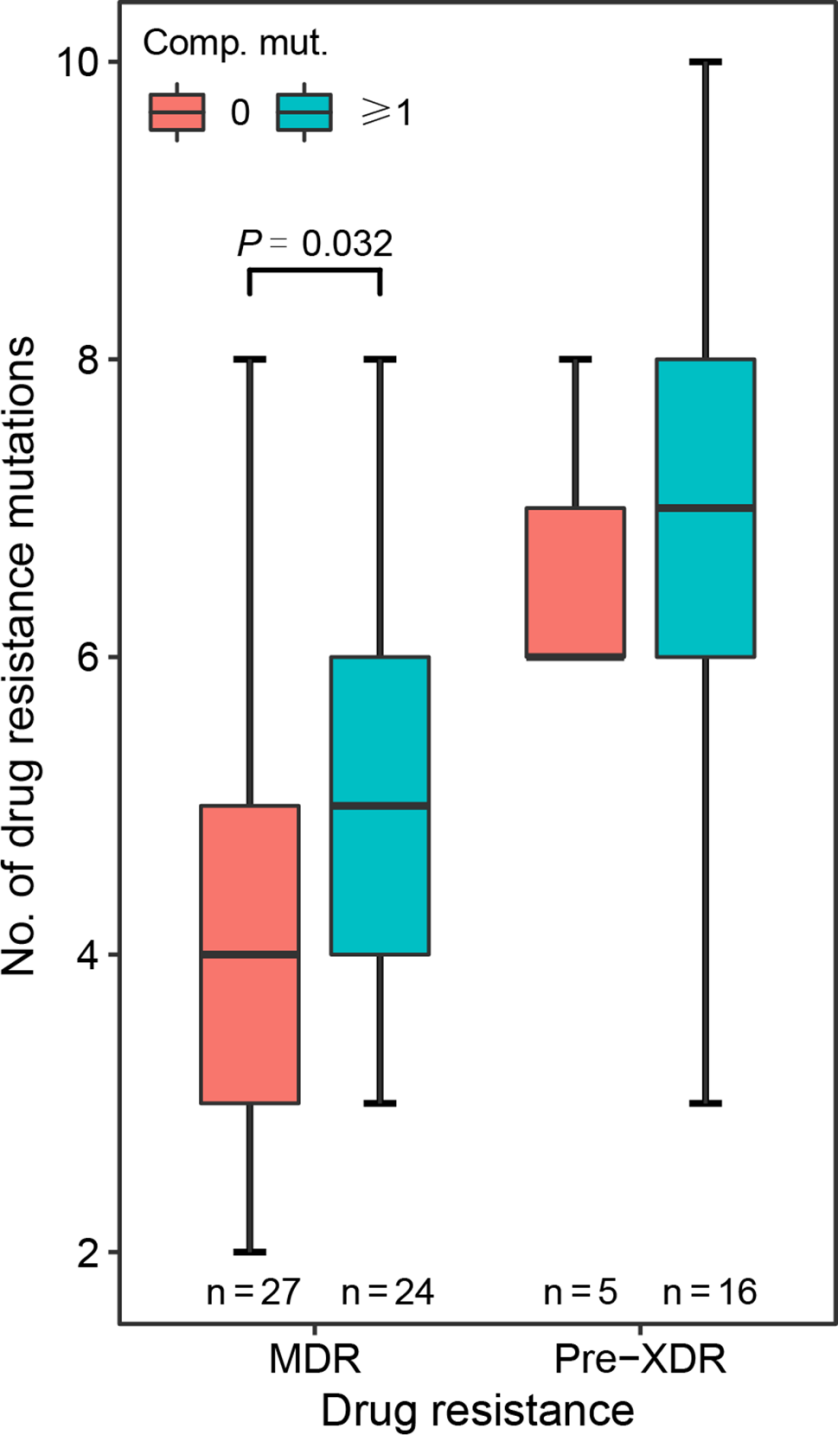
Distribution of drug resistance variants stratified by compensatory mutations. Comp. mut., compensatory mutations

**Fig. 7:**
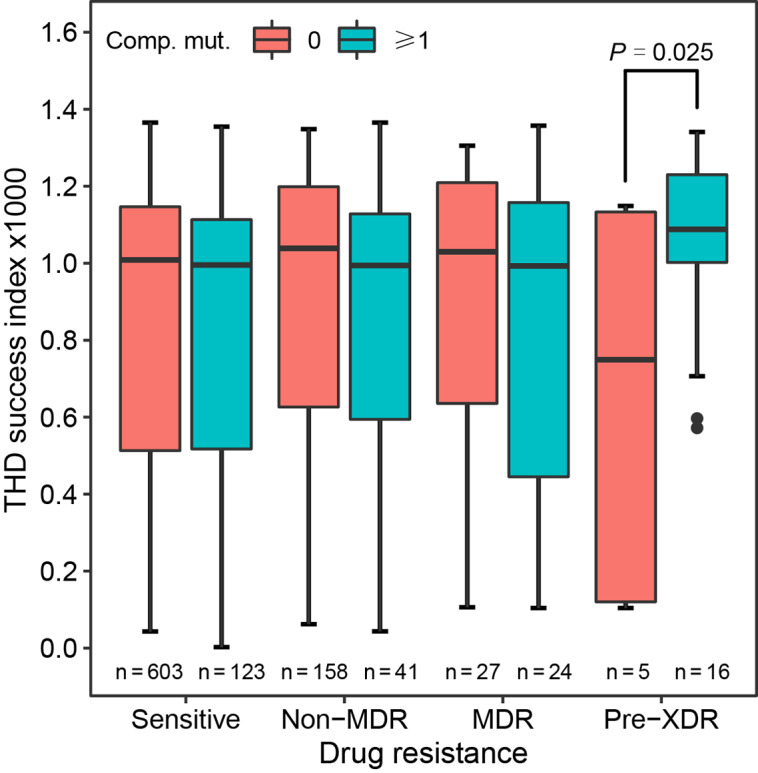
Relationship between drug resistance, compensatory mutations, and THD success index. Comp. mut., compensatory mutations

Then, we assessed the relationship between antibiotic resistance and compensatory mutations, as well as their influence on the epidemic success of *Mtb* strains. Interestingly, the results found the highest prevalence of compensatory mutation in pre-XDR strains (76.19%), followed by MDR (47.06%), non-MDR (20.60%), and sensitive (16.94%) strains (Table 1). Subgroup analyses showed a higher level of resistance-associated mutations in MDR strains with compensatory mutations (*P* = 0.032), while a similar finding was not detected for pre-XDR strains (*P* = 0.524) (Fig. 6). Notably, given the small sample size (n = 21), further research is warranted to testify the above findings concerning pre-XDR strains. For isolates with different drug resistance, the THD success indices were found to be similar (*P* = 0.340). To disentangle the respective impacts of drug resistance and fitness compensation on the transmission of *Mtb* isolates, subgroup analyses were further implemented. The findings suggested that pre-XDR strains with compensatory mutations had higher success indexes than those without compensatory mutations (*P* = 0.025), of which the medians were 1.09 (IQR, 1.00-1.23) and 0.75 (IQR, 0.12–1.13), respectively (Fig. 7).

Furthermore, we evaluated the transmission status of *Mtb* isolates across regions, and found strains circulating in Zhejiang Province had a higher success index compared to those in Shanghai City (median, 1.03 versus 0.72; IQR, 0.64–1.16 versus 0.11–1.14; *P* < 0.001).

### Genetic factors for *Mtb* isolates success

To further identify the genetic factors accounting for the epidemic success of lineage 2 and lineage 4, we performed homoplasy analysis to find identical mutations that independently occurred in parallel branches of phylogenetic trees. Those mutations were possibly under positive selection and cannot be explained by phylogenetic evolution [24]. Of 3333 mutations, 85 were located in genes indicating drug resistance and fitness compensation among lineage 2. Similarly, 24 mutations were found for lineage 4 strains, with a total of 693 sites under positive selection (Supplementary Table 2). The remaining mutations in non-canonical or unclear association with drug resistance or compensatory effects [29–31] occurred in 1569 genes and 317 genes among lineage 2 and lineage 4, respectively, including 5 chromosomal loci in *whiB6* gene associated with a high rate of drug resistance to second-line injectables [30].

Besides, there were seven and one non-synonymous mutations in *Rv1129c* (*prpR*) gene among lineage 2 and lineage 4, respectively, which had been proved to induce conditional drug tolerance by altering the propionate metabolism of *Mtb* strains [32]. Dating analyses suggested local *Mtb* isolates acquired *prpR* gene mutation (D160A) around 51 (95% HPD, 42–81) years before present. Additionally, we also detected mutations in *dnaA* gene for both lineage 2 and lineage 4, which were recently reported to increase resistance to isoniazid [33].

## Discussion

In this study, a large-scale WGS-based analysis involving 997 *Mtb* isolates from eastern China was performed to clarify the distribution and evolution of *Mtb* lineages, as well as genetic drug resistance. Furthermore, we also attempted to explore the epidemic transmission of isolates and relevant genomic factors using THD method and homoplasy analyses. The results suggested L2.2 was the dominant strain circulating in eastern China (80.34%), followed by L4.4 (8.93%) and L4.5 (8.43%). The proportion of lineage 2 in eastern China (80.44%) was higher than that in Yunnan Province (70.47%) [34] and Xinjiang Autonomous Region (57.48%) [35], but lower than that in Heilongjiang Province (89.5%) [36], indicating a geography-specific distribution of *Mtb* lineages.

Previous studies indicated human migration may impact the transmission of *Mtb* isolates [7, 12]. In this study, coalescent-based Bayesian evolution analyses suggested an estimated epidemic time of approximately 7000 years for lineage 2 in eastern China, slightly longer than that of lineage 4 (nearly 6900 years). The transmission of lineage 2 and lineage 4 might be related to the development of agricultural civilization in the middle and lower reaches of the Yangtze River, given the similar timing of the events and geographic location [37]. Furthermore, we detected an obvious population expansion of lineage 4 around 2200 years ago, which was probably fueled by the march of *Qin’*s army aiming to conquer the territory of *Chu* and *Baiyue* States (comprising partial central region and most eastern region of China) during the same period [38]. As one of the widely distributed *Mtb* lineages, lineage 4 is deemed to originate from Europe, followed by a spread to the American continent [39]. In China, lineage 4 was reported to prevail mainly in the western regions [40], but the isolates in eastern China have been experiencing ongoing transmission for the past two centuries according to our findings, consistent with the ending time of isolationist policies of *Qing* Dynasty. Meanwhile, this historical episode also facilitated the spread of lineage 2, which was further supported by our findings. Nowadays, the features of population migration in China are from rural to urban areas and from inland to coastal regions [41], and meanwhile, economic globalization entails more international exchanges. In such a context, human mobility help facilitate bacterial transmission to the eastern region of China. Similarly to our findings, a recent study [22] also demonstrated a rapid population growth of lineage 4 in Zhejiang Province. Thus, although lineage 2 is the predominating lineage in eastern China, it is necessary to pay sufficient attention to lineage 4 given its increased population size.

In this study, the highest resistance rate was observed for isoniazid (15.25%), followed by streptomycin (12.34%) and rifampicin (8.93%). Of 152 HR-TB strains, 127 (83.55%) harbored mutations in *katG* gene, and isolates with *katG* S315T mutation accounted for 66.45%. A systematic review involving 11,411 *Mtb* isolates concluded that *katG*315 mutation contributed to 64% of phenotypic isoniazid resistance, approximating the frequency of this mutation in our study [42]. Besides, the prevalence of mutations in *rpsL* and *rrs* genes among streptomycin-resistant isolates were found to be 69.11% (85/123) and 8.94% (11/123), which were similar to prior reports from southwest China (76.11% and 7.2%) [43], but different from those found in Iran (both 36.8%) [44]. Over one-half of streptomycin-resistant isolates harbored *rpsL*43 mutation (66/123), suggesting a major contribution to the high level of streptomycin resistance by such mutations. For RR-TB isolates, the most frequent drug-resistant mutation was *rpoB* S450L (50.56%), similar to the findings of Zhou et al. [45]. Furthermore, we also found 2.11% of strains were pre-XDR based on the new definition of drug resistance [6], accounting for 29.17% of MDR strains. The overall rate of fluoroquinolone resistance was found as 6.12%, the highest rate of drug resistance to second-line anti-TB drugs. As resistance to fluoroquinolones is revealed to be a main risk factor for the failure of treatment for MDR-TB [46, 47], the high prevalence of fluoroquinolone resistance in this study may potentially impair the efficacy of MDR-TB regimens and increase transmission risk. In addition, this study explored the dynamic and temporal accumulation of mutations conferring drug resistance and revealed an earlier emergence of mutations against isoniazid and rifampicin, while mutations conferring fluoroquinolone resistance appeared relatively later, consistent with the observed order of resistance acquisition in a global study [48]. This finding can be partially explained by the order of drug administration, as fluoroquinolones will be only administrated after resistance to first-line drugs is detected.

Lineage 2 has been frequently reported to be associated with drug resistance [49, 50] and treatment failure [51]. But when compared to lineage 4 (23.59%) in this study, there was no significantly higher drug resistance rate for lineage 2 (28.05%). Similar to our findings, Yuan et al. [52] reported 26.73% of Beijing lineage isolates developed drug resistance and 24.69% in non-Beijing lineage isolates, and the prevalence of MDR-TB in both groups was also comparable (6.91% versus 5.56%). Besides, a seven-year population-based cohort study in northern Malawi also demonstrated similar drug resistance patterns between Beijing and non-Beijing genotypic strains [53]. The discordance in the association of lineages with drug resistance across studies might be due to different proportions of lineage 2 strains circulating in local populations [54]. Considering these inconsistent findings, relevant factors for the discrepancy of drug resistance across lineages should be elucidated by further research, which is suggested to consider both social-economic and genomic aspects.

By far, few studies explored the epidemic success of *Mtb* isolates and relevant factors at an individual level. In the current study, THD method was employed to carry out further analyses. The epidemicity between lineage 2 and lineage 4 strains was comparable, and we did not observe an obvious difference in transmission capacity between sensitive, MDR, and pre-XDR strains, but the findings revealed the crucial roles of compensatory mutations for the transmission of pre-XDR strains in eastern China. In line with our findings, Wu et al. [22] reported a similar and increasing trend of effective sample size for L2.2, L4.2, L4.4, and L4.5 in China, suggesting analogous transmission capacity between *Mtb* lineages. Glynn et al. [53] also reported *Mtb* isolates with Beijing genotype did not increase the severity or transmissibility of TB, compared to non-Beijing genotypic isolates. The skyline plot in this study also showed the prevalence of lineage 4 in recent years exhibited an increasing trend like lineage 2. By contrast, Yang et al. [55] proposed lineage 2 favors the epidemic expansion of *Mtb* isolates. It might be due to the case that *Mtb* transmission can be impacted by multiple factors, which vary in different geographic settings [56]. Overall, the evidence suggests lineage 2 and lineage 4 have equivalent transmission risks and should be given equal and enough attention. Furthermore, we detected a bimodal distribution of THD indices for L2.2, of which isolates with low transmission capacity cluster with those with high transmission capacity, indicating a complex transmission pattern of local *Mtb* isolates.

Drug resistance helps to facilitate the spread of *Mtb* isolates, as well as to induce fitness cost, which in turn contributes to slow bacterial growth and transmission risk [57, 58]. To reduce these adverse effects, drug-resistant *Mtb* isolates can acquire secondary, compensatory mutations to partially or fully restore the fitness cost. In this study, we found the acquisition of compensatory mutations after introduction of canonical mutations conferring resistance to isoniazid (*katG* S315T) and rifampicin (*rpoB* S450L), suggesting a potential role in impacting the epidemic success of *Mtb* strains. Consistent with these results, we also detected the proportion of isolates with compensatory mutations increased with the degree of resistance from sensitive, MDR to pre-XDR in this study. Additionally, more drug resistance-associated mutations were detected for MDR strains with compensatory mutations, and pre-XDR strains with compensatory mutations had a higher success index compared to their counterpart without such mutations. Interestingly, we did not find a lower transmission capacity of pre-XDR strains than other drug-resistant isolates. We presumed that compensatory mutations in pre-XDR strains may result in the mitigation of fitness cost due to drug resistance, thereby inducing similar transmissibility compared to isolates with other drug resistance. Besides, as mentioned above, the epidemic efficiency of *Mtb* isolates can be influenced by TB control programs, treatment regimens, characteristics of local population, and social-economic conditions, etc. [56]. Therefore, the correlation of drug resistance with bacterial transmission may simply reflect the local TB epidemic rather than the intrinsic properties of drug-resistant strains.

In this study, we also carried out homoplasy analysis to identify SNP under positive selection contributing to epidemic success. Interestingly, most positively selected sites were in genes that were not canonically correlated with drug resistance, partially explaining the null association of drug resistance with the bacterial transmission. Compared to the findings in Mumbai [5] and the European/Russian region [12], we revealed a larger number of positively selected sites for *Mtb* isolates in eastern China, suggesting a more common phenomenon of homoplasy in local population. The occurrence of homoplasic sites does not result from inheritance at the hierarchical level, and they arise independently in different branches of phylogenetic trees under selection pressure [59]. Consequently, homoplasy can lead to convergent population phenotypes in adaptive evolution, which are conducive to the survival of *Mtb* isolates [60]. Furthermore, in addition to canonical mutations conferring drug resistance, our results offered further evidence that several allelic sites under positive selection contribute to increased drug tolerance, like *whiB6, prpR*, and *dnaA*, which can be regarded as potential genetic markers to infer drug resistance.

However, several limitations of the presented study should be pointed out. First, as samples were collected in Zhejiang Province and Shanghai City, potential selection bias should be under consideration when interpreting our findings despite a certain degree of representation for local isolates circulating in eastern China. Second, the estimated emergence time of drug-resistant mutations was conservative, since we traced the common ancestor of resistance clades, rather than the time for the acquisition of mutations themselves. Third, we did not further explore the association of genetic factors with the epidemic success of *Mtb* isolates using multivariate correlational analysis adjusting for confounders, like pulmonary infection and socioeconomic factors [11, 56], because these data were not available in the current study. Briefly, our study can offer further evidence about the population phylogeny and emergence time of genetic drug resistance in *Mtb* isolates in eastern China, as well as the genetic background underlying the bacterial transmission.

## Conclusions

In conclusion, this study indicates clonal expansions of lineage 2 and lineage 4 in eastern China, with similar transmission capacity. There is a chronological accumulation of resistance mutations in *Mtb* strains, while which do not impose significant facilitation effects on the epidemic. Furthermore, we revealed a positive correlation between drug resistance and compensatory mutations, which help facilitate the epidemiological transmission of pre-XDR isolates. Despite a lower prevalence compared to lineage 2, the epidemic of lineage 4 in eastern China should call for considerable attention given its increased population size in recent years [46, 47]. As pre-XDR strains are fluoroquinolone-resistant, a risk factor for treatment failure, prospective molecular surveillance is suggested to monitor the evolution of pre-XDR/XDR strains, thereby taking timely interventions.

## Electronic supplementary material

Below is the link to the electronic supplementary material.

Supplementary Material 1

Supplementary Material 2

Supplementary Material 3

## Data Availability

All data in this study can be obtained from the website of National Center for Biotechnology Information (BioProject accession number: PRJNA874331).

## Abbreviations

ESS: Effective sample size
HPD: Highest posterior density
HR: Isoniazid-resistant
MCMC: Markov chain Monte Carlo
MDR: Multidrug-resistant
ML: Maximum-likelihood
*Mtb*: *Mycobacterium tuberculosis*
RR: Rifampicin-resistant
TB: Tuberculosis
THD: Time-scaled haplotypic density
tMRCA: Time to the most recent common ancestor
WGS: Whole genome sequencing
