# Supplementary material for: Evolution and epidemic success of Mycobacterium tuberculosis in eastern China: evidence from a prospective study
Source: BMC Genomics. 2023 May 5;24:241. doi: 10.1186/s12864-023-09312-6 (PMC10161668; doi:10.1186/s12864-023-09312-6)
Supplement: Supplementary file 1 — Supplementary Material 1 [file 12864_2023_9312_MOESM1_ESM.docx]

**
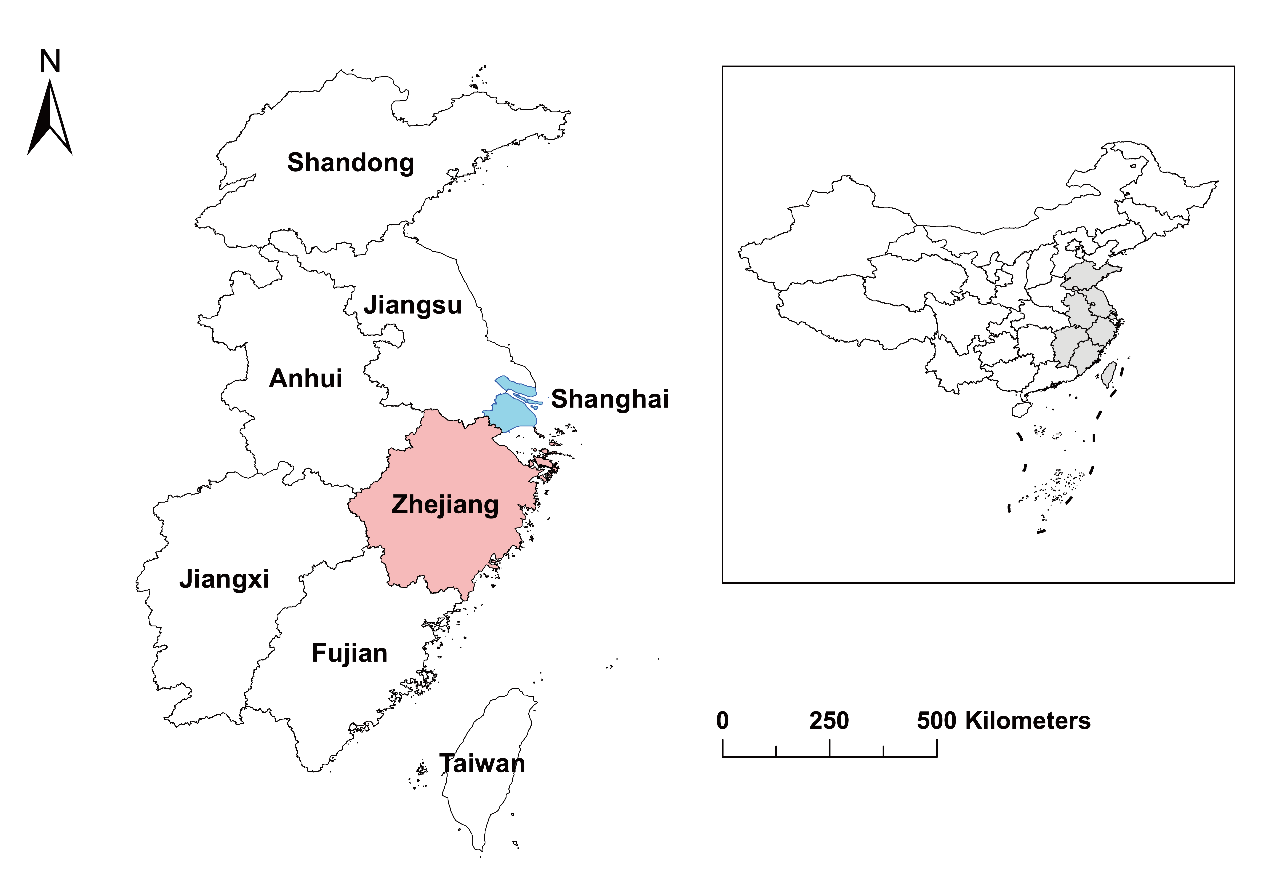
**

**Fig. S1** Map showing the location of study area

**
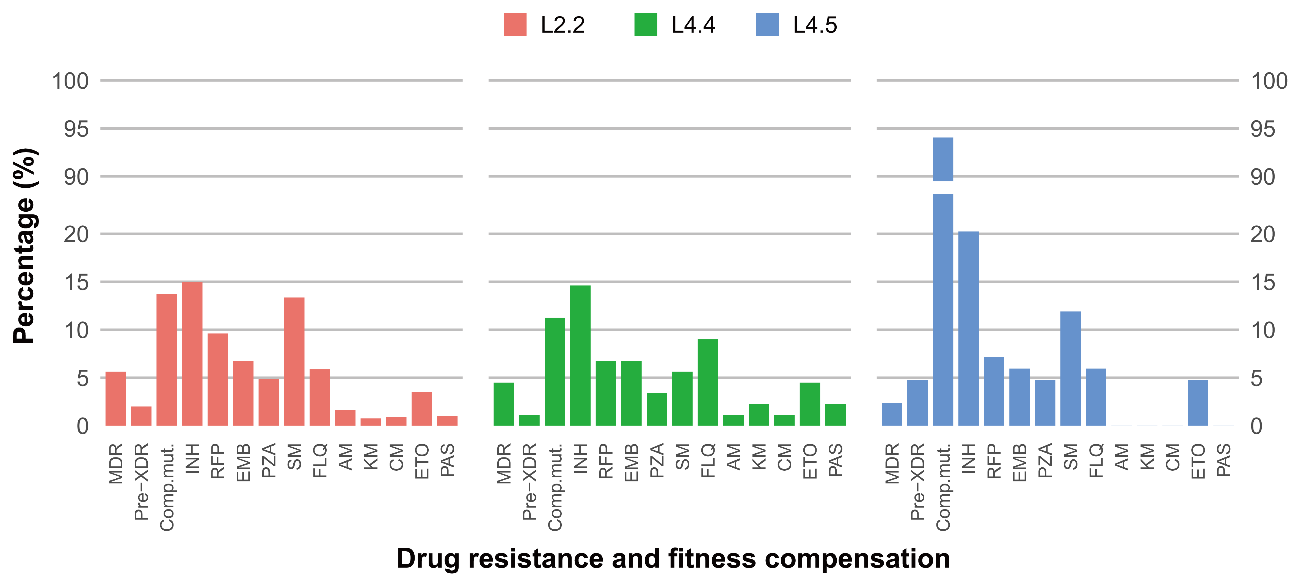
**

**Fig. S2** Drug resistance and compensatory mutations in L2.2, L4.4 and L4.5. INH, isoniazid; RFP, rifampicin; Comp. mut., compensatory mutations; EMB, ethambutol; PZA, pyrazinamide; SM, streptomycin; FLQ, fluoroquinolones; AM, amikacin; KM, kanamycin; CM, capreomycin; ETO, ethionamide; PAS, para-aminosalicylic acid
